# Digital Rehabilitation for Acute Ankle Sprains: Prospective Longitudinal Cohort Study

**DOI:** 10.2196/31247

**Published:** 2021-09-30

**Authors:** Fernando D Correia, Maria Molinos, Carlos Neves, Dora Janela, Diana Carvalho, Sara Luis, Gerard E Francisco, Jorge Lains, Virgilio Bento

**Affiliations:** 1 Neurology Department Centro Hospitalar e Universitário do Porto Porto Portugal; 2 Escola de Medicina Universidade do Minho Braga Portugal; 3 SWORD Health Technologies, Inc Draper, UT United States; 4 Department of Physical Medicine and Rehabilitation McGovern Medical School The University of Texas Health Science Center Houston, TX United States; 5 TIRR Memorial Hermann Houston, TX United States; 6 Rovisco Pais Medical and Rehabilitation Centre Tocha Portugal; 7 Faculty of Medicine Coimbra University Coimbra Portugal

**Keywords:** acute ankle sprains, physical rehabilitation, home-based digital rehabilitation, digital therapy, rehabilitation, sprain, digital health, therapy, rehabilitation, prospective, longitudinal, cohort, ankle, soft tissue, physical therapy, pain, outcome, fatigue

## Abstract

**Background:**

Ankle sprains are one of the most prevalent soft-tissue injuries worldwide. Physical therapy, especially progressive exercise, has proven effective in improving function, while preventing recurrence.

**Objective:**

We aim to present the results of a fully remote and digitally guided rehabilitation program for acute ankle sprains.

**Methods:**

We performed a prospective longitudinal cohort study of individuals eligible for workers’ compensation, who were referred for digital rehabilitation therapy for a sprained ankle. Therapeutic exercise sessions were to be performed independently by the patient at home using the biofeedback device provided by SWORD Health. Primary endpoints were the change in self-reported Numerical Pain Rating Scale (NPRS) and Foot and Ankle Ability Measure–activities of daily living (FAAM–ADL) and FAAM–Sports scores. Participants were assessed at baseline, end of the program, and 6 months after program completion. Secondary outcomes included digital therapy dosage, pain and fatigue during sessions, and satisfaction.

**Results:**

In total, 93 (89.4%) patients completed the program and 79 (76.0%) were available for follow-up. Changes in the primary outcomes between baseline and the 6-month follow-up were both significant (*P*<.001) and clinically meaningful: mean difference of –2.72 points (95% CI –3.31 to –2.13) on the NPRS (49.8% reduction), 21.7 points (95% CI 17.13-26.27) on the FAAM–ADL (41.1% increase), and 37.8 points (95% CI 30.45-45.15) on the FAAM-Sports (151.8% increase). Longer waiting periods between the accident date and treatment initiation were found to negatively impact functional status at baseline and at the end of the program, triggering an extension in the program duration. The total training volume (12.5 hours, SD 10.5 hours) was similar to that of other interventions for ankle sprains, but the dosage per week was much higher (2.4 hours per week, SD 0.87 hours per week). The mean patient satisfaction score was 8.8 (SD 1.57) out of 10. Among program completers, 83.9% attained full recovery and were discharged with no residual disability.

**Conclusions:**

Being far less demanding in terms of human resources, the digital program presented constituted a viable, clinically effective, and convenient solution for ankle sprain rehabilitation, particularly during the pandemic. This is the first study presenting a fully remote home-based rehabilitation program for acute ankle sprains, with patients achieving sustained long-term results. This was a prospective cohort study and, as such, did not include a control group, but the results appear comparable to those published for face-to-face interventions.

**Trial Registration:**

ClinicalTrials.gov NCT04819022; https://clinicaltrials.gov/ct2/show/NCT04819022

## Introduction

Ankle sprains are one of the most prevalent soft-tissue injuries, with an estimated incidence of 2.15 per 1000 person-years in the United States [1] and 5-7 per 1000 person-years in Europe [2]. They are more common in the second and third decades of life [3], but only about half are associated with sports participation, suggesting that they may affect individuals with different physical activity levels [1].

Given their high incidence, ankle sprains have an important socioeconomic impact, mainly from indirect costs [4,5]. Overall costs range from US $1809-$5271 per patient, with direct costs representing US $292-$2268 [6]. Other studies estimate that indirect costs make up for 70%-90% of the total costs [5,7].

It has also been observed that 12%-47% of all ankle sprains are recurrent [8-12], and at least one-third of individuals experience residual symptoms [13-15]. In fact, evidence suggests that individuals with previous ankle sprain are at an approximately 3.5 times greater risk of recurrence [1], and that up to 45% of patients report an incomplete recovery 3 years after injury [16].

Ensuring complete recovery and a decreased risk of reinjury are therefore of paramount importance. Physical therapy, especially progressive exercise, has been shown to not only improve function [17-23] but also prevent recurrence [17,24,25]. Effectiveness seems to improve with intensity, especially in doses of more than 900 minutes of total exercise time [18].

Notwithstanding, access to physical therapy interventions remains a challenge, owing to physical mobility and transportation limitations [26-29]. Home-based interventions have been studied as alternatives. However, despite being associated with improved outcomes [30], a systematic review reported diminished gains in pain and physical capacities when compared to supervised rehabilitation [31]. Additionally, low compliance is a known issue [32].

A potential solution is telerehabilitation, which helps alleviate time, travel, and access barriers, while potentiating intensity and satisfaction [33-36]. Another advantage of telerehabilitation is the minimal person-to-person contact, which is particularly relevant in the actual pandemic context. Indeed, there is growing research on its application in a variety of musculoskeletal conditions [33,34,37-39], with promising results in comparison with conventional care [35,40]. Studies have also demonstrated that remote patient assessment is technically feasible and valid for ankle joint disorders [36]. However, there is still a lack of adoption of telerehabilitation [41] as well as intrinsic limitations regarding access to technology and the need for real-time availability of a physical therapist.

To overcome this limitation, technological approaches allowing independent home-based rehabilitation have been developed, but these are still experimental, and clinical validation is scant [42]. In previous clinical studies, we demonstrated the feasibility and safety of digitally delivered rehabilitation programs after total knee and hip replacement [43-45], as well as the ability to maximize clinical outcomes over conventional physical therapy through the same technology.

New digital programs aimed at treating other conditions have since been developed. This paper presents the results of a prospective, consecutive cohort of patients undergoing a fully remote rehabilitation program for acute ankle sprain.

## Methods

### Study Design

This is a prospective, longitudinal cohort study aimed to assess the clinical outcomes of digital rehabilitation programs provided by SWORD Health.

### Participants

Individuals eligible for workers’ compensation under health plans, which have entered into a commercial agreement with SWORD Health, acting as an in-network provider of physical therapy services, were recruited. Patients were initially assessed by their orthopedic surgeon and referred for physical therapy after confirmation of an ankle sprain (based on clinical and imaging findings). Referral to in-network providers of physical therapy was managed administratively, with the possibility of explicit referral to the digital program by the orthopedic surgeon.

### Rehabilitation Program

The plan of care was based on therapeutic exercise sessions to be performed independently by the patient at home using the biofeedback device provided by SWORD Health, in accordance with the protocol presented in Multimedia Appendix 1. Patients were instructed to perform 1 exercise session per day. The plan was adapted by the treating physician in articulation with the physical therapist as needed. Program duration and patient discharge were determined by the treating physician.

### Study Outcomes

The primary outcome was the change in the self-reported Numerical Pain Rating Scale (NPRS) score (0-10), as well as the change in the Foot and Ankle Ability Measure (FAAM) [46]—for activities of daily living (FAAM–ADL) and sports (FAAM–Sports) between baseline and the 6-month follow-up. Patients were assessed for these outcomes at baseline, end of the rehabilitation program, and at 6 months, through an electronic survey. The NPRS was self-reported at each assessment survey with the question, “How would you rate your pain over the last 7 days – from 0 (no pain at all) to 10 (worst pain imaginable)?”

Secondary outcomes included the following user experience–related outcomes, collected along the program by the digital therapist:

1. Treatment dosage: program duration (days), number of sessions per week, total number of sessions, minutes per session, and total exercising time (minutes and hours).
2. Average pain during sessions: self-reported at the end of each session; visual analogue scale (VAS) scores of 0-10;
3. Change in pain during sessions: last versus first VAS score for pain registered;
4. Average fatigue during sessions: self-reported at the end of each session; VAS scores of 0-10;
5. Change in fatigue during sessions: last versus first VAS score for fatigue registered;
6. Satisfaction: assessed at the end of the program with the question, “On a scale from 0 to 10, how likely is it that you would recommend this intervention to a friend or neighbor?”

### Statistical Analysis

To assess differences in primary and secondary outcomes among the 3 time points, a Bonferroni multiple comparison test was performed with time as a categorical variable. Both unadjusted and adjusted differences for covariates with 95% CIs were estimated. Included covariates were age, gender, BMI, days to start treatment, grade of sprain, exercise level, and previous injury.

Linear mixed-effects models (LMM) were also utilized to assess participant change across NPRS, FAAM–ADL, and FAAM–Sports metrics from baseline to the end of the study. This type of model was chosen over a repeated-measures analysis of variance (ANOVA) since the former allows for a relaxation of model assumptions (ie, it does not assume that variances and covariances among groups are equal), a more flexible treatment of time (which is treated as a continuous variable and not a category), and makes it easier to include covariates (as additional fixed-effects) [47].

For each outcome, a model was created without including covariates (unadjusted) and including covariates (adjusted). Multiple imputation using 50 imputed data sets was used to account for attrition in each variable across time [48].

A bivariate correlation analysis was also performed to investigate covariates’ association with outcomes.

A repeated-measures ANOVA was also performed for the primary outcomes with time as the within-subjects factor and grade of sprain (grades I-III per the guidelines of Lynch [49]) as the between-subjects factor. The same approach was used with program duration categories (<4 weeks and >4 weeks) as the between-subjects factor.

LMM analysis was performed using R. All the other statistical analyses were performed using SPSS (version 17.0; SPSS Inc).

### Data Availability

Deidentified individual participant data are provided in Multimedia Appendix 2.

## Results

### Baseline Characteristics of Participants

**Figure 1:**
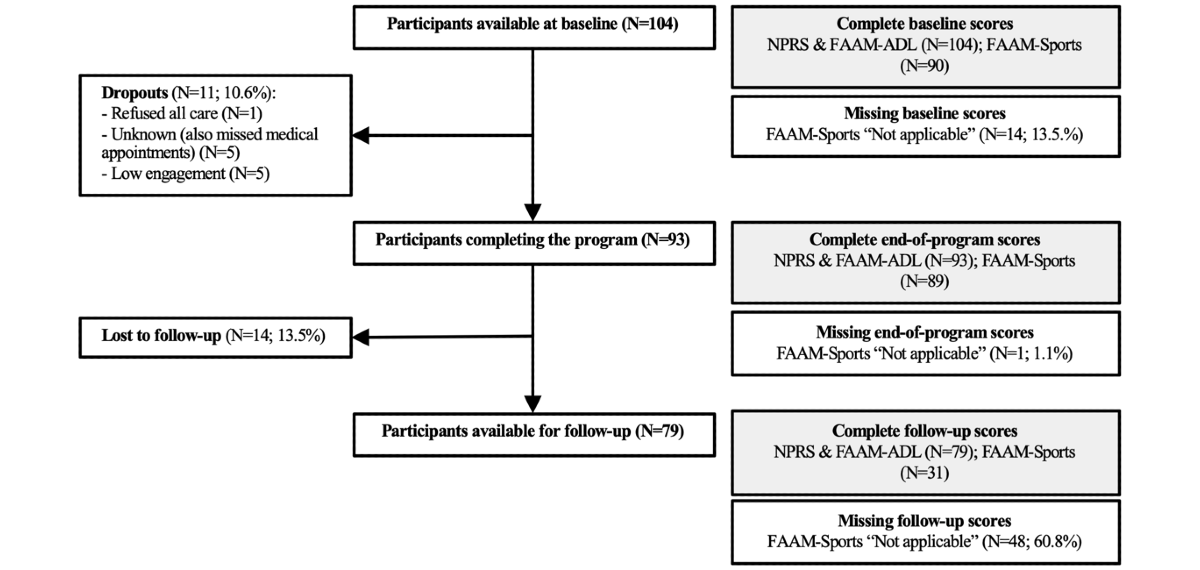
Flow chart for participant inclusion (left) and attrition (right). FAAM–ADL: Foot and Ankle Ability Measure–activities of daily living, FAAM–Sports: Foot and Ankle Ability Measure–sports, NPRS: Numerical Pain Rating Scale.

In total, 104 patients from 4 different recruitment sites and 26 different orthopedic surgeons were consecutively enrolled in SWORD Health’s fully remote physical therapy program for ankle sprain between February and November 2020 (Figure 1). The dropout rate was 10.6% (11/104); 1 (1.0%) patient subsequently refused all types of care proposed by the physician, 5 (4.8%) dropped out owing to unknown reasons (missing exercise sessions and medical appointments), and 5 (4.8%) did not adhere to the digital program. In total, 93 (89.4%) patients completed the program, and 79 (76.0%) were available for follow-up.

Participant’s baseline characteristics are presented in Table 1. Baseline assessment of the outcome variables is summarized in Table 2.

**Table 1:** Baseline characteristics of participants who finished the rehabilitation program (N=93).

Characteristic		Value
Age (years), mean (SD)		40.7 (10.43)
**Age (years), n (%)**		
	<25	9 (9.7)
	25-40	36 (38.7)
	>40	48 (51.6)
Females, n (%)		50 (53.8)
BMI, mean (SD)		27.8 (4.98)
**BMI categories, n (%)**		
	Underweight (<18.5)	0 (0)
	Normal (18.5-25)	32 (34.4)
	Overweight (25-30)	32 (34.4)
	Obese (>30)	29 (31.2)
Affected side: right, n (%)		47 (50.5)
**Grade of ankle sprain, n (%)^a^**		
	I	53 (57.0)
	II	27 (29.0)
	III	10 (10.8)
**Exercise level (hours per week), n (%)**		
	0	36 (38.7)
	1-2	30 (32.3)
	3-4	16 (17.2)
	≥5	11 (11.8)
Previous injury, n (%)		27 (29)
Previous surgery, n (%)		0
Time from injury date to treatment initiation (days), mean (SD)		53.2 (48.26)
Time from referral to SWORD and treatment initiation (days), mean (SD)		3.8 (2.17)

^a^Three observations are missing.

**Table 2:** Estimates for patient-reported outcomes at baseline, end of the program, and 6-month follow-up assessment, unadjusted and adjusted for covariates.

Time		Unadjusted for covariates, mean (95% CI)	Adjusted for covariates^a^, mean (95% CI)
**Numerical Pain Rating Scale score**			
	Baseline	5.67 (5.16-6.17)	5.46 (4.86-6.05)
	End of program	3.67 (3.16-4.17)	3.46 (2.87-4.06)
	6-month follow-up	2.92 (2.42-3.43)	2.73 (2.15-3.32)
**Foot and Ankle Ability Measure–activities of daily living score**			
	Baseline	52.00 (47.90-56.10)	52.70 (47.80-57.60)
	End of program	63.50 (59.50-67.60)	64.30 (59.50-69.20)
	6-month follow-up	73.50 (69.50-74.40)	74.40 (69.60-79.20)
**Foot and Ankle Ability Measure–sports score**			
	Baseline	26.70 (20.40-32.90)	24.90 (17.40-32.40)
	End of program	46.20 (39.90-52.40)	44.60 (37.20-52.00)
	6-month follow-up	64.30 (58.00-70.50)	62.70 (55.30-70.10)
**Visual analogue scale for pain score (0-10)**			
	Baseline	3.98 (3.56-4.40)	3.52 (2.74-4.30)
	End of program	2.84 (2.37-3.31)	2.36 (1.88-2.85)
**Visual analogue scale for fatigue score (0-10)**			
	Baseline	2.73 (2.30-3.17)	2.08 (1.29-2.87)
	End of program	2.77 (2.25-3.30)	2.15 (1.61-2.69)

^a^Adjusted for age, gender, BMI, days to start treatment, grade of sprain, exercise level, and previous injury.

### Longitudinal Changes in Outcomes

Table 2 presents the primary and secondary clinical outcomes. All patients showed significant improvement in NPRS, FAAM–ADL, FAAM–Sports, and VAS pain scores (*P*<.001) from baseline to 6-month follow-up (Table 3).

Essentially, considering the minimal clinically important difference values established for FAAM subscales [50] (ie, 8 points for FAAM–ADL and 9 points for FAAM–Sports), the registered changes from baseline were also clinically meaningful. Overall, patients experienced reductions of 50% and 33% in the NPRS and VAS pain scores, respectively, which can also be considered clinically significant.

**Table 3:** Differences in adjusted^a^ estimates upon multiple comparison at different time points assessed for the primary endpoints, pain, and fatigue sessions.

Time-point comparisons		Estimate difference (95% CI)	SE	*t* test (*df*)	Bonferroni *P* value
**Numerical Pain Rating Scale score**					
	End of program vs baseline	–1.99 (–2.58 to –1.40)	0.30	–6.63 (*184*)	<.001^b^
	6-month follow-up vs baseline	–2.72 (–3.31 to –2.13)	0.30	–9.04 (*184*)	<.001^b^
	6-month follow-up vs end of program	–0.73 (–1.32 to –0.14)	0.30	–2.42 (184)	.05^b^
**Foot and Ankle Ability Measure–activities of daily living score**					
	End of program vs baseline	11.60 (7.03 to 16.17)	2.33	5.00 (*184*)	<.001^b^
	6-month follow-up vs baseline	21.70 (17.13 to 26.27)	2.33	9.31 (*184*)	<.001^b^
	6-month follow-up vs end of program	10.00 (5.43 to 14.57)	2.33	4.31 (*184*)	<.001^b^
**Foot and Ankle Ability Measure–sports score**					
	End of program vs baseline	19.70 (12.37 to 27.03)	3.74	5.26 (*184*)	<.001^b^
	6-month follow-up vs baseline	37.80 (30.45 to 45.15)	3.75	10.10 (*184*)	<.001^b^
	6-month follow-up vs end of program	18.10 (10.77 to 25.43)	3.74	4.84 (*184*)	<.001^b^
**Visual analogue scale for pain score (0-10)**					
	End of program vs baseline	–1.16 (–1.64 to –0.67)	0.24	–4.72 (*89*)	<.001^b^
**Visual analogue scale for fatigue score (0-10)**					
	End of program vs baseline	0.07 (–0.47 to 0.61)	0.28	0.24 (*89*)	.81

^a^Adjusted for age, gender, BMI, days to start treatment, grade of sprain, exercise level, and previous injury.

^b^Statistically significant at *P*<.05.

**Figure 2:**
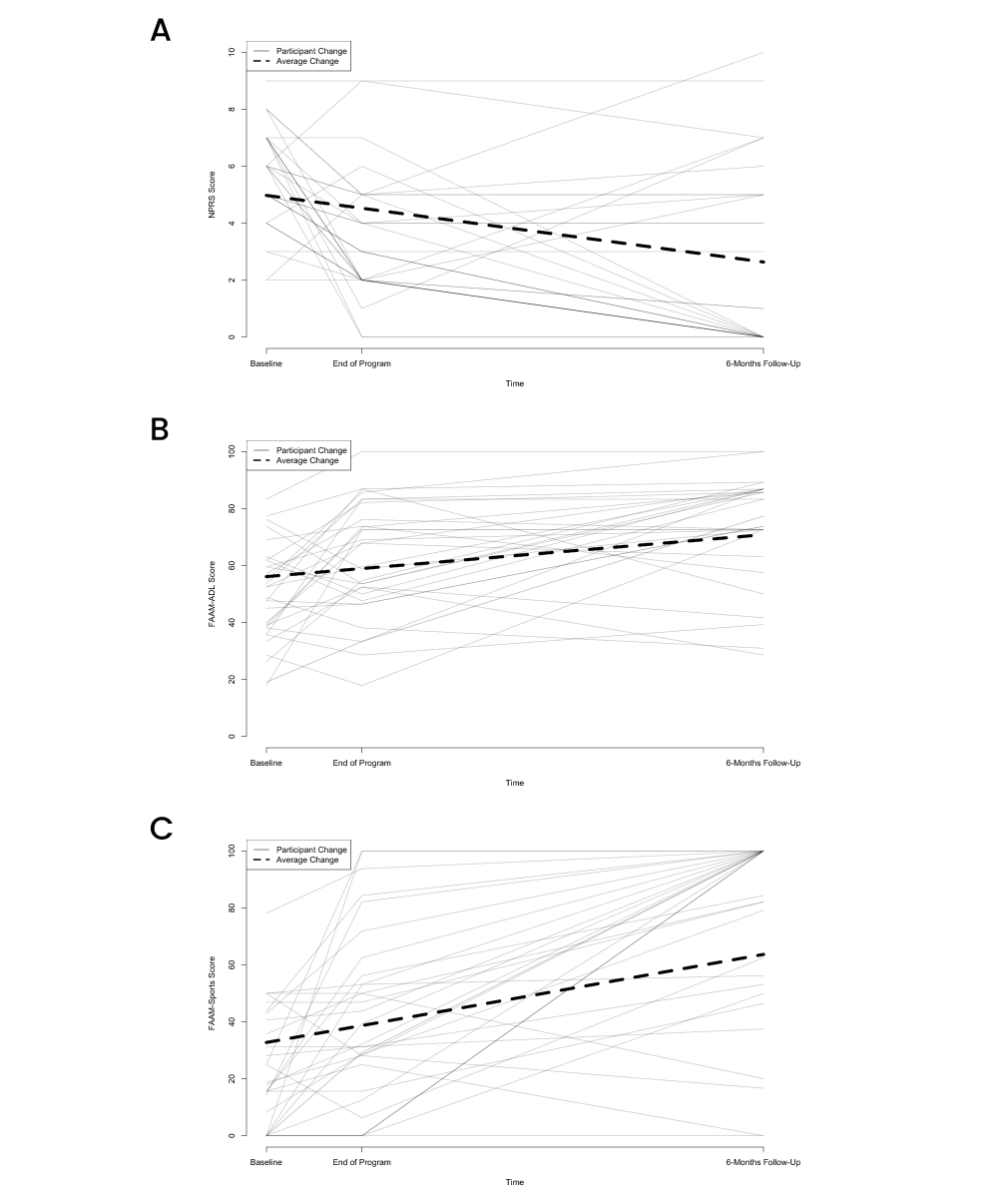
Linear mixed model showing the individual and aggregate longitudinal changes in the primary endpoints (FAAM–ADL, FAAM–Sports, and NPRS scores). Each thin line represents a participant, and the thick dotted line represents the average change across all participants. Covariates appearing in the model include age, gender, BMI, days to start treatment, grade of sprain, exercise level, and previous injury. Significant effects were found for covariates (*P*<.05). FAAM–ADL: Foot and Ankle Ability Measure–activities of daily living, FAAM–Sports: Foot and Ankle Ability Measure–sports, NPRS: Numerical Pain Rating Scale.

LMM analysis with 50 imputed data sets revealed that for all unadjusted models, significant effects of time were observed in the expected directions. This indicates that over the larger study timeline, participants reported significant improvements in NPRS, FAAM–ADL, or FAAM–Sports scores (Multimedia Appendix 3). Longitudinal changes in ankle function and pain perception are depicted in Figure 2.

Similar effects of time were found for all adjusted models (Multimedia Appendix 3). Regarding NPRS scores, participants with a higher average BMI (estimate=0.08; *P*=.04) and those who took days to start treatment (estimate=0.01; *P*=.02) had significantly higher scores. A significant negative linear effect of sprain grade was also observed (estimate=–1.00; *P*=.02). No covariates significantly affected FAAM–ADL scores. Regarding FAAM–Sports scores, older participants showed significantly lower scores (estimate=0.58; *P*=.03). A significant negative quadratic effect of sprain grade was also observed (estimate=0.01; *P*=.02).

### Usability-Related Outcomes

#### Compliance and Training Intensity

Program completers performed on average 5.9 (SD 1.34, range 0.9-7.8) sessions per week (Table 4), with 39.8% (37/93) of them performing 7 sessions per week, 45.2% (42/93) performing 5-6 sessions per week, and 15% (14/93) performing less than 5 sessions per week. The mean program duration was 5.0 (SD 3.81, range 0.9-19.6) weeks, and the mean total exercise dosage was 750.6 (SD 630.25, range 77.1-2836.4) minutes, with 30% (28/93) of patients executing more than 900 minutes of exercise therapy, a threshold that has been described as being associated with better outcomes [18]. No significant correlation was found between total exercising time and primary endpoints.

#### Session-Related Pain and Fatigue

On their last session, patients reported significantly lesser pain than the initial session (*P*<.001). Patient-reported fatigue did not change (*P*=.81; Table 3), which reflects progression of session intensity/difficulty over time. Mean values for VAS pain and VAS fatigue scores during exercise sessions are presented in Table 4.

**Table 4:** System usability–related outcomes for patients who finished the rehabilitation program (N=87).

Usability outcomes	Measure	
	Mean (SD)	Range
Program duration (weeks)	5.0 (3.81)	0.9-19.6
Sessions per week	5.9 (1.34)	0.9-7.8
Total number of sessions	28.9 (21.99)	3-115
Total exercising time (min)	750.6 (630.25)	77.1-2836.4
Total exercising time (hours)	12.5 (10.50)	1.3-47.3
Minutes per session	24.7 (5.86)	12.0-37.2
Average pain during sessions (visual analogue scale for pain score, 0-10)	3.5 (1.45)	0 -7.7
Average fatigue during sessions (visual analogue scale for fatigue score, 0-10)	3.1 (1.66)	0-6.8
Satisfaction (0-10)^a^	8.8 (1.57)	4-10

^a^Two observations are missing.

#### Satisfaction

The mean satisfaction score was 8.8 (SD 1.57, range 4-10) points (Table 4). In total, 63.4% (59/93) of patients answered “9” or “10,” 23.7% (22/93) answered between “7” and “8,” and 10.7% (10/93) answered “6” or less.

### Disability and Return to Work

Among those who finished the program, 83.9% (78/93) of patients were classified by the treating physician as having obtained maximum medical improvement and no residual disability, while 12 (13%) patients were rendered with permanent partial disability, 2 (2%) with temporary total disability, and 1 (1%) with permanent total disability.

In total, 48.4% (45/93) of completers returned to work before clinical discharge. Within those discharged with no residual disability, the majority (53.8%, 42/78) did not return to work before clinical discharge, likely because of the shorter treatment period (median 2.8 weeks, IQR 4.04 weeks; range 0.86-9.57 weeks).

### Reinjury and Adverse Events

The reinjury rate was 2.5%; 2 of the 79 available patients for follow-up reported ankle sprain recurrence as a result of a fall. One was a patient who had had 4 previous ankle sprains, and the other did not have a history of previous injury. Both had been discharged with no residual disability.

The adverse event rate was 3.2% (Multimedia Appendix 4). One patient reported exacerbated ankle pain caused by long walks; one could not perform the sessions due to a suspected plantar fasciitis; and another reported intense lower back pain, limiting compliance with the exercise protocol. None were related to the digital intervention.

### Subgroup Analysis

#### Grade of Ankle Sprain

**Figure 3:**
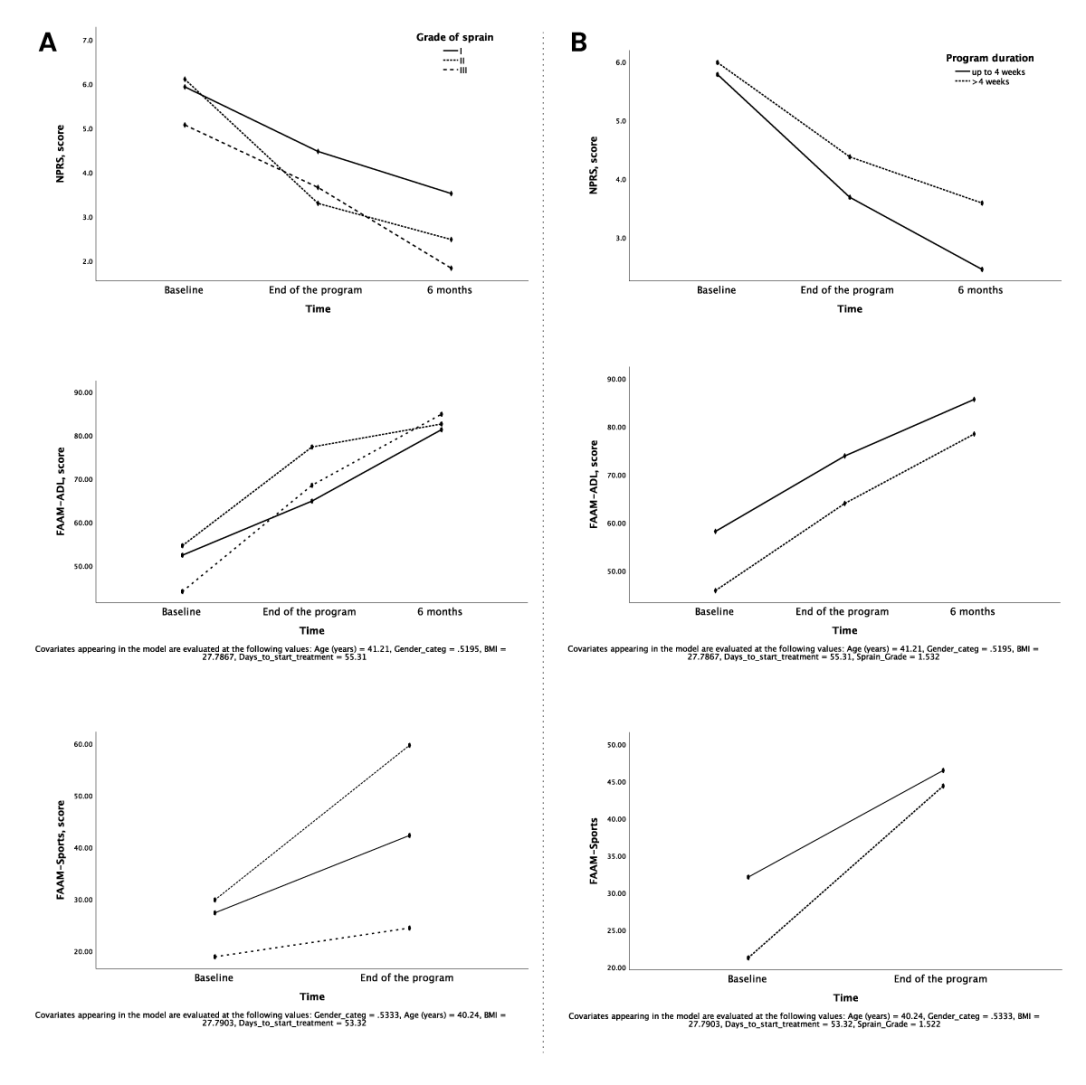
Estimated marginal means over time based on (A) sprain grade I, II , and III for NPRS and FAAM–ADL scores (n=45, 23, and 9, respectively; baseline to 6-month follow-up) and for FAAM–Sports (n=47, 24, and 7, respectively; baseline to the end of the program) and (B) program duration up to 4 weeks and above 4 weeks for NPRS and FASAM–ADL scores (n=39 and 38, respectively; baseline to 6-month follow-up), and for FAAM–Sports (n=41 and 37, respectively; baseline to the end of the program). FAAM–ADL: Foot and Ankle Ability Measure–activities of daily living, FAAM–Sports: Foot and Ankle Ability Measure–sports, NPRS: Numerical Pain Rating Scale.

This analysis confirmed a main effect of time for the 3 dimensions of NPRS (*P*=.05) and FAAM–ADL (*P*=.003) after 6 months. The results are presented in Tables 5 and 6 and Figure 3.

**Table 5:** Repeated-measures analysis of variance for the primary outcomes based on the grade of sprain.

Outcome variable	Grade of sprain					
	Time		Sprain grade		Time × sprain grade	
	*F* test (*df*1, *df*2)	*P* value	*F* test (*df*1, *df*2)	*P* value	*F* test (*df*1, *df*2)	*P* value
Numerical Pain Rating Scale score	3.30 (1.77, 123.74)	.05	2.22 (2, 70)	.12	1.22 (3.54, 123.74)	.31
Foot and Ankle Ability Measure–activities of daily living score	6.15 (1.90, 133.21)	.003	0.91 (2, 70)	.41	1.84 (3.81, 133.21)	.13
Numerical Pain Rating Scale score	4.58 (1, 83)	.03	3.63 (2, 83)	.03	4.15 (2, 83)	.02

**Table 6:** Repeated-measures analysis of variance for the primary outcomes based on the duration of the program.

Outcome variable	Program duration					
	Time		Program duration		Time × program duration	
	*F* test (*df*1, *df*2)	*P* value	*F* test (*df*1, *df*2)	*P* value	*F* test (*df*1, *df*2)	*P* value
Numerical Pain Rating Scale score	2.31 (1.80, 126.07)	.11	3.03 (1, 70)	.09	0.98 (1.80, 126.07)	.37
Foot and Ankle Ability Measure–activities of daily living score	3.70 (1.89, 131.98)	.03	7.62 (1, 70)	.01	0.50 (1.88, 131.98)	.60
Numerical Pain Rating Scale score	3.97 (1, 83)	.05	1.43 (1, 83)	.24	2.16 (1, 83)	.14

Overall, patients with sprain grade II experienced the greatest improvement, with outcomes converging in the long term.

Regarding FAAM–Sports scores, it was not possible to account for the 6-month follow-up in the repeated-measures analysis, since 60.8% (48/79) of answers to the FAMM–Sports questionnaire were not applicable during the pandemic period, yielding a very small sample size per subgroup (n=18, 10, and 3 for sprain grades I, II, and III, respectively). Outcomes following the end of the program for this dimension revealed a main effect of time (*P*=.05) and grade of sprain (*P*=.03) and an interaction between time and grade of sprain (*P*=.02). Differences among the 3 subgroups were detected at the end of the program (*P*=.01, 1-way ANOVA), with post hoc multiple comparisons showing that patients with grade II sprain scored significantly higher than those with grade III sprain (*P*=.01; mean difference 34.1 points, 95% CI 6.28-61.94 points).

#### Program Duration

The mean program duration was 5.0 (SD 3.81, range 0.9-19.9; median 4.1, IQR 5.0; 95% CI 4.3-5.8) weeks, with over half of the sample (54.8%, 51/93) discharged within 4 weeks. Hence, a cut-off of 4 weeks was established to explore differences in outcomes between patients discharged before or after that cut-off.

This analysis confirmed a main effect of time and program duration for FAAM–ADL after 6 months (*P*=.03 and *P*=.01, respectively). An effect of time was also observed on FAAM–Sports (*P*=.05) between baseline and the end of the program. No other effects or interactions were detected (Table 5 and Figure 3).

Patients requiring >4 weeks of treatment had significantly worse baseline and end-of-program FAAM–ADL scores (*P*=.002 and *P*=.02, respectively; independent samples *t* test). NPRS was not different at baseline (*P*=.76), but patients in the <4 weeks group reported less pain at the 6-month follow-up assessment (*P*=.05; independent samples *t* test) along with better functional outcomes (*P*=.03 for FAAM–ADL; independent samples *t* test). FAMM–Sports scores were also not different at baseline (*P*=.05; independent samples *t* test), and patients in both subgroups recovered similarly for this dimension.

## Discussion

### Principal Findings

This study shows that a fully remote, home-based, digital rehabilitation program for acute ankle sprains delivered at patients’ homes allowed patients to attain clinically meaningful improvement in pain (evident from their VAS and NPRS scores), activities of daily living (FAAM–ADL scores), and sports activities (FAAM–Sports scores). Furthermore, these programs led to a full recovery without residual disability in 83.9% of patients, which compares favorably with the published literature showing that at least one-third of individuals will experience residual symptoms [13-15].

There is a dearth of studies on digital programs for acute ankle sprains, as supported by recently published systematic [51] and literature [52] reviews on the subject. We therefore broadened the search to include exercise-based approaches in general [23,53-55]. Overall, the results obtained in this study are similar to those reported for other supervised exercise programs, and the first detailed positive outcomes with a fully digital program.

One RCT (n=90) [55] assessed the effectiveness of exercise training using the Nintendo Wii Fit balance board in comparison to physical therapy and to a control receiving no therapy. Investigators found this tool was not more effective than PT only or no exercise. Of note, patients enrolled in this study had little room left for improvement, with near-normal scores at baseline on the FAAM–ADL (mean 71-83) and FAAM–Sports (mean 37-52), and low VAS pain (approximately mean 1 point), which may have been the reason behind no difference between physical therapy only or no exercise.

In another RCT (n=74) [54] comparing an manual therapy and exercise (MTEX) program with a home exercise program (HEP), the improvement in the MTEX program at 4 weeks was similar to what we observed in this study: FAAM–ADL score, mean 21.3 (95% CI 18.2-24.5) points; FAAM–Sports score, mean 27.1 (95% CI 22.7-31.6) points; and NPRS score, mean –2.7 (95% CI –2.9 to –2.5) points. When compared to the HEP group, our intervention also provided superior outcomes in terms of functional recovery and pain.

Both NPRS baseline values and its magnitude of change from baseline to the end of the program were similar to the ones reported for other exercise interventions after ankle sprain [23,54,56].

### Recurrence and Completeness of Recovery

This study corroborates previous findings of high recurrence rates both among nonathlete (24%-54%) [57,58] and athlete (12%-47%) populations, with 29% of all enrolled patients having had previous injury.

Also consistent with our findings, the group from Verhagen found that a home-based proprioceptive 8-week training program, delivered through a mobile app after usual care, was successful in reducing recurrences of ankle sprains in a 12-month period as against conventional care alone (22% versus 33%, as revealed through an RCT with 522 athletes from the Netherlands) [59]. Although the rate of reinjury was still much higher than that reported here at 6 months (2.5%), this further supports the effectiveness of remote interventions in preventing ankle reinjuries.

Previous findings indicate an association between the rate of resprain and incomplete recovery [57]. Therefore, the high percentage of complete recovery attained in this study may explain the lower rate of recurrence, even if the 2 patients who experienced recurrence had been discharged with no residual disability. In fact, by the 6-month follow-up, 45.6% (36/79) and 35.5% (11/31) of patients in this study, respectively, achieved scores compatible with the normative values for FAAM–ADL and FAAM–Sports reported for the adult population (92.3 and 85.1 points, respectively) [60].

### Training Volume

In a systematic review and meta-analysis, Bleakley et al [61] found no clear consensus on an optimal training volume, with rehabilitation times ranging from 3.5 to 21 hours (median 12 hours). The highest total rehabilitation time was 21 hours, equivalent to 1.75 hours per week over 12 weeks. In our study, the mean total exercising time was 12.5 (SD 10.50, range 1.3-47.3) hours, equivalent to 2.4 (SD 0.87, range 0.4-4.6) hours per week. Hence, the total training volume was similar to that of other interventions, but dosage per week was much higher.

### Subgroup Outcomes

Even though overall changes from baseline to follow-up were not significantly different between patients discharged before or after 4 weeks (no interaction found between time and program duration), the latter patients had worse FAAM–ADL scores both prior to participating in the program and at discharge, and worse NPRS and FAAM–ADL scores at 6 months.

We hypothesize this could be a consequence of the particularly long period between the injury date and treatment initiation—mean 53.2 (SD 48.26, range 4-281) days—mainly in relation to disruptions in health care delivery in the wake of the COVID-19 pandemic. (ie, a delay between injury and the physician appointment) (Table 1). Indeed, we found a correlation between longer waiting periods and extended program duration (Pearson *r*_93_=0.48; *P*<.001), with mean waiting times of 40.9 (SD 38.79) days for patients who were discharged within 4 weeks versus 68.1 (SD 54.57) days for those discharged after 4 weeks (*P*=.01).

Recent reviews have not found sufficient evidence regarding independent predictors of clinical outcomes [62,63]. Only 1 study so far gathered proof that a low injury grade is a predictor for better outcomes [64].

In this study, no differences were found in terms of program duration between injury grades (*P*=.11, 1-way ANOVA; grade I: 4.8 weeks, 95% CI 3.8-5.8 weeks; grade II: 4.7 weeks, 95% CI 3.2-6.2 weeks; grade III: 7.5 weeks, 95% CI 3.7-11.2 weeks). Nonetheless, we found differences between injury grade and clinical improvement. Patients with grade II injuries experienced the greatest improvement during the program, followed by those with grade I and then grade III injuries. This could be explained by the fact that grade I sprains are only directed to physical therapy in case of aggravation, at a point where they actually become slower respondents. In the long term, however, patients with grade III sprain reported the greatest improvement in NPRS and FAAM–Sports scores, followed by those with grade II and grade I sprain, as confirmed by LMM analysis. This is most likely related to the lower FAAM scores and higher pain levels at baseline in patients with grade III sprain, consequently with a higher margin of progression. These aspects, along with the convergence of clinical outcomes over time for the 3 groups, do not support the notion of a low injury grade being a predictor for better outcomes.

### Limitations

The limitations of this study are mainly related to the study design and the referral process. This was a prospective cohort study and, as such, did not include a control group. However, as shown above, our results are comparable to those reported previously for supervised exercise programs in this same context. Regarding the referral process, while patient assignment to an in-network provider was largely performed administratively, explicit referral to digital programs was possible. This may have introduced a selection bias toward patients more likely to engage in digital care.

### Conclusions

This was the first study presenting the outcomes from a fully remote exercise-based rehabilitation program for acute ankle sprains, demonstrating clinically meaningful change in both pain and function, as well as complete recovery in 81.7% of patients, with sustained results over time. As such, this study demonstrates not only the feasibility of fully digital programs in this context, but also that these programs can achieve clinical outcomes comparable to face-to-face interventions.

## Appendix

Multimedia Appendix 1 Fully remote rehabilitation protocol applied for acute ankle sprains.

Multimedia Appendix 2 De-identified participants data.

Multimedia Appendix 3 Support statistical analysis tables.

Multimedia Appendix 4 Adverse events list.

## Abbreviations

ANOVA: analysis of variance
FAAM–ADL: Foot and Ankle Ability Measure–activities of daily living
FAAM–Sports: Foot and Ankle Ability Measure–sports
HEP: home exercise program
LMM: linear mixed-effects model
MTEX: manual therapy and exercise
NPRS: Numerical Pain Rating Scale
RCT: randomized controlled trial
VAS: visual analogue scale
