# Supplementary material for: Digital Rehabilitation for Acute Ankle Sprains: Prospective Longitudinal Cohort Study
Source: JMIR Rehabil Assist Technol. 2021 Sep 30;8(3):e31247. doi: 10.2196/31247 (PMC8517823; doi:10.2196/31247)
Supplement: Multimedia Appendix 1 [file rehab_v8i3e31247_app1.docx]

**Rehabilitation Protocol**

**Table 1.1S**

**Sub-Acute Phase**

***Week 1 and 2***

| **Sub-Acute Phase** | | |
| --- | --- | --- |
| **Main Goals** | | |
| To be able to walk without crutches  Decrease the pain level  Increase the asymptomatic range of motion  Protect the surrounding structures  Prevent muscle strength loss | | |
| **Intervention**  *Daily Digital Therapist Sessions*  *Remote session – Assessment/Deploy* | | |
| **Digital Therapist Exercises** | | **Mobility Session** |
| **Standing** | Hip flexion w/ knee flexion (with ankle dorsiflexion) (bilateral) | 2 sets of 12 repetitions (alternating) |
|  | Hip abduction (with ankle dorsiflexion) (bilateral) | 2 sets of 12 repetitions (alternating) |
| **Sitting** | Knee flexion (with ankle dorsiflexion) (unilateral) | 2 sets of 10 repetitions (alternating) |
|  | Knee extension (with ankle dorsiflexion) (unilateral) | 2 sets of 10 repetitions (alternating) |
| **Lying** | Knee flexion (unilateral) | 2 sets of 10 repetitions (alternating) |
|  | *Clamshells* | 2 sets of 10 repetitions (alternating) |
|  | Bridge | 2 sets of 10 repetitions (alternating) |
| **Estimated Training Time** | | **20 min** |

**Table 1.2S**

**Functional rehabilitation phase**

***Week 3***

| **Functional Rehabilitation Phase** | | |
| --- | --- | --- |
| **Main Goals** | | |
| Decrease pain (0/10 VAS)  Normalize range of motion  Normalize muscular strength  Normalize functionality  Return to daily activities | | |
| **Intervention**  *Daily Digital Therapist Sessions* | | |
| **Digital Therapist Exercises** | | **Mobility Session** |
| **Standing** | Hip Flexion W/ Knee Flexion (With Ankle Dorsiflexion) (Bilateral) | 2 set of 12 repetitions (alternating) |
|  | Hip Abduction (With Ankle Dorsiflexion) (Bilateral) | 2 set of 12 repetitions (alternating) |
|  | Forward Lunge (Bilateral) | 2 set of 12 repetitions (alternating) |
|  | Side Lunge (Unilateral) | 2 set of 12 repetitions (alternating) |
|  | Plantar Flexion (Bilateral) | 2 set of 12 repetitions (alternating) |
|  | Airplane (Unilateral) | 2 set of 12 repetitions (alternating) |
|  | Squat | 2 set of 12 repetitions (alternating) |
| **Lying** | *Clamshells* | 2 sets of 12 repetitions (alternating) |
|  | Unilateral Bridge (Unilateral) | 2 set of 12 repetitions (alternating) |
| **Estimated Training Time** | | **28 min** |

**Table 1.3S**

**Functional rehabilitation phase**

***Week 4***

| **Functional Rehabilitation Phase** | | |
| --- | --- | --- |
| **Goals** | | |
| Decrease pain (0/10 VAS)  Normalize range of motion  Normalize muscular strength  Normalize functionality  Return to daily activities | | |
| **Intervention**  *Daily Digital Therapist Sessions* | | |
| **Digital Therapist Exercises** | | **Mobility Session** |
| **Standing** | Hip Flexion w/ Knee Flexion (With Ankle Dorsiflexion) (Bilateral) | 1 set of 20 repetitions (alternating) |
|  | Hip Abduction (With Ankle Dorsiflexion) Contralateral | 1 set of 12 repetitions (alternating) |
|  | Forward Lunge (Bilateral) | 2 set of 12 repetitions (alternating) |
|  | Backward Lunge (Bilateral) | 2 set of 12 repetitions (alternating) |
|  | Side Lunge (Unilateral) | 2 set of 12 repetitions (alternating) |
|  | Airplane (Unilateral) | 2 set of 8 repetitions (alternating) |
|  | Pistols (Unilateral) | 2 set of 12 repetitions (alternating) |
|  | Plantar Flexion (Unilateral) | 2 set of 12 repetitions (alternating) |
| **Lying** | *Clamshells* | 2 sets of 12 repetitions (alternating) |
|  | Unilateral Hamstring Bridge | 2 set of 12 repetitions (alternating) |
| **Estimated Training Time** | | **29 min** |
