# Supplementary material for: Digital Rehabilitation for Acute Ankle Sprains: Prospective Longitudinal Cohort Study
Source: JMIR Rehabil Assist Technol. 2021 Sep 30;8(3):e31247. doi: 10.2196/31247 (PMC8517823; doi:10.2196/31247)
Supplement: Multimedia Appendix 3 [file rehab_v8i3e31247_app3.docx]

**Statistical Tables**

Linear mixed-effects models were utilized to assess participant change across NPRS, FAAM ADL, and FAAM sports metrics from baseline to the end of the study. This type of model is known as a growth curve analysis^[[1]](#footnote-1)^. For each outcome, a model was created without including covariates (unadjusted) and including covariates (adjusted). All unadjusted models were of the form:

$$y_{ij}=\gamma_{00}+\gamma_{10}T+u_{0i}+u_{1i}T+e_{ij}$$

where $y_{ij}$ is the value of a survey at time *i* for participant *j*, $\gamma_{00}$ is a fixed-effect representing the average baseline score of each participant, $T$ is a time variable in weeks (0, 5, 26) representing change in $y_{ij}$ baseline (0), from the end of the study (5), or at a follow-up measurement (26), $\gamma_{10}$ is a fixed-effect representing average expected time slope from baseline across all participants, $u_{0j}$ is a random-effect representing individual participant deviation from the average baseline score (i.e, a random-intercept term), $u_{1j}$ is a random-effect representing individual participant deviation from the average change over time (i.e, a random-slope term), and $e_{ij}$ represents an error term.

Table 3.1S represents the results of the unadjusted linear model on each outcome variable.

| Table 3.1S: Unadjusted LMM Results. | | | | | | |
| --- | --- | --- | --- | --- | --- | --- |
| DV | Variable | Fixed-Effect (95%-CI) | Random-Effect (SD) | MI-statistic | df | *P* |
| NPRS | **Intercept** | **4.97 (4.57, 5.38)** | **1.21** | **23.94** | **270.59** | **< .001** |
|  | **Slope** | **-0.09 (-0.11, -0.07)** | **0.04** | **-7.30** | **236.55** | **< .001** |
| FAAM-ADL | **Intercept** | **56.11 (52.22, 60)** | **11.93** | **28.29** | **211.69** | **< .001** |
|  | **Slope** | **0.57 (0.29, 0.84)** | **0.36** | **3.99** | **68.41** | **< .001** |
| FAAM-sports | **Intercept** | **32.74 (27.53, 37.95)** | **18.95** | **12.31** | **266.21** | **< .001** |
|  | **Slope** | **1.19 (0.76, 1.62)** | **1.12** | **5.42** | **84.85** | **< .001** |
| ***p* < .05** |  |  |  |  |  |  |

On average NPRS scores decreased from baseline to the 6-month follow-up at a rate of -0.09 (-0.11, -0.07) units per week, MI-statistic = -7.30; *P* < .001. FAAM-ADL scores on average increased by 0.57 (0.29, 0.84) units per week, MI-statistic = 3.99; *P* < .001. Finally, FAAM-Sports scores on average increased 1.19 (0.76, 1.62) units per week, MI-statistic = 5.42; *P* < .001.

Table 3.2S represents the results of the covariate adjusted linear model on each outcome variable. Age and BMI have been centered. Female is binary (0,1) representing the participant being either male (0) or female (1). Sprain grade was modeled as an ordered categorical variable (Sprain Grade I, II, or III) with possible linear and quadratic effects. Sports hours was also modeled as an ordered categorical variable (1-2, 3-4, 4-5, 5 or more).

| Table 3.2S: Covariate Adjusted LMM Results. | | | | | | |
| --- | --- | --- | --- | --- | --- | --- |
| DV | Variable | Fixed-Effect (95%-CI) | Random-Effect (SD) | MI-statistic | df | *P* |
| NPRS | **Intercept** | **4.03 (3.22, 4.84)** | **1.205** | **9.76** | **256.18** | **< .001** |
|  | **Slope** | **-0.09 (-0.11, -0.07)** | **0.043** | **-7.29** | **227.08** | **< .001** |
|  | Age | 0.03 (0.00, 0.07) |  | 1.71 | 251.51 | .089 |
|  | Female | 0.24 (-0.50, 0.98) |  | 0.64 | 251.97 | .521 |
|  | **BMI** | **0.08 (0.00, 0.15)** |  | **2.07** | **253.33** | **.039** |
|  | **Sprain Grade - L** | **-1.00 (-1.86, -0.13)** |  | **-2.26** | **221.22** | **.025** |
|  | Sprain Grade - Q | -0.03 (-0.78, 0.72) |  | -0.08 | 206.31 | .933 |
|  | **Days to Start** | **0.01 (0.00, 0.02)** |  | **2.37** | **254.25** | **.019** |
|  | Sports Hours - L | 0.34 (-0.54, 1.21) |  | 0.75 | 256.74 | .455 |
|  | Sports Hours - Q | 0.13 (-0.68, 0.94) |  | 0.31 | 255.25 | .758 |
|  | Sports Hours - C | -0.13 (-0.91, 0.66) |  | -0.32 | 254.44 | .752 |
|  | Previous Injury | 0.12 (-0.68, 0.93) |  | 0.30 | 255.52 | .762 |
| FAAM-ADL | **Intercept** | **60.88 (53.22, 68.54)** | **12.54** | **15.58** | **171.89** | **< .001** |
|  | **Slope** | **0.57 (0.29, 0.84)** | **0.37** | **3.98** | **66.93** | **< .001** |
|  | Age | -0.1 (-0.48, 0.29) |  | -0.50 | 130.47 | .619 |
|  | Female | -2.85 (-9.99, 4.28) |  | -0.78 | 145.17 | .435 |
|  | BMI | -0.12 (-0.83, 0.59) |  | -0.32 | 143.15 | .749 |
|  | Sprain Grade - L | -1.14 (-9.42, 7.13) |  | -0.27 | 127.91 | .787 |
|  | Sprain Grade - Q | -6.51 (-13.8, 0.79) |  | -1.75 | 111.03 | .083 |
|  | Days to Start | -0.08 (-0.15, 0) |  | -1.95 | 153.26 | .053 |
|  | Sports Hours - L | -3.15 (-11.65, 5.36) |  | -0.73 | 147.30 | .469 |
|  | Sports Hours - Q | -6.95 (-14.7, 0.8) |  | -1.76 | 155.74 | .081 |
|  | Sports Hours - C | -3.82 (-11.48, 3.85) |  | -0.98 | 140.76 | .331 |
|  | Previous Injury | 0.69 (-7.02, 8.39) |  | 0.18 | 152.11 | .861 |
| FAAM-sports | **Intercept** | **35.14 (24.6, 45.68)** | **20.64** | **6.54** | **236.46** | **< .001** |
|  | **Slope** | **1.19 (0.76, 1.62)** | **1.13** | **5.42** | **83.49** | **< .001** |
|  | **Age** | **-0.58 (-1.08, -0.07)** |  | **-2.23** | **215.02** | **.027** |
|  | Female | 0.82 (-8.7, 10.34) |  | 0.17 | 232.38 | .867 |
|  | BMI | -0.27 (-1.25, 0.7) |  | -0.55 | 205.02 | .582 |
|  | Sprain Grade - L | -3.5 (-15.11, 8.11) |  | -0.59 | 167.68 | .555 |
|  | **Sprain Grade - Q** | **-11.83 (-21.88, -1.78)** |  | **-2.31** | **154.17** | **.022** |
|  | Days to Start | -0.08 (-0.18, 0.03) |  | -1.46 | 221.85 | .147 |
|  | Sports Hours - L | -5.07 (-16.53, 6.39) |  | -0.87 | 227.43 | .387 |
|  | Sports Hours - Q | -4.78 (-15.24, 5.68) |  | -0.90 | 237.10 | .372 |
|  | Sports Hours - C | -3.78 (-14.32, 6.76) |  | -0.70 | 202.13 | .483 |
|  | Previous Injury | -1.04 (-11.57, 9.48) |  | -0.19 | 222.83 | .846 |
| ***p* < .05** |  |  |  |  |  |  |

1. McArdle, J. J., & Nesselroade, J. R. (2003). Growth curve analysis in contemporary psychological research. *Handbook of psychology*, 447-480. [↑](#footnote-ref-1)
