# Supplementary material for: Digital Rehabilitation for Acute Ankle Sprains: Prospective Longitudinal Cohort Study
Source: JMIR Rehabil Assist Technol. 2021 Sep 30;8(3):e31247. doi: 10.2196/31247 (PMC8517823; doi:10.2196/31247)
Supplement: Multimedia Appendix 4 [file rehab_v8i3e31247_app4.docx]

**Adverse events list**

Supplemental Table 4S: Type and description of adverse events registered during the rehabilitation program.

| **Adverse event type** | **Nº cases** | **Adverse event description** | **Resolved?** | **Medication?** | **Medical assistance?** |
| --- | --- | --- | --- | --- | --- |
| Mild | 1 | exacerbated ankle pain caused by long walks | Yes | No | No |
| Moderate | 1 | plantar fasciitis | Yes | Yes | No |
|  | 1 | low back pain due to a left sacroiliac blockage | Yes | Yes | Yes |
| **Total** | 3 |  |  |  |  |
